# Gluten‐free diet management and well‐being in children with celiac disease: A qualitative study

**DOI:** 10.1111/pai.70061

**Published:** 2025-03-18

**Authors:** Heather Maddison‐Roberts, Christina Jones, Rose‐Marie Satherley

**Affiliations:** ^1^ School of Psychology, Department of Psychological Interventions, Faculty of Health and Medical Sciences University of Surrey Guildford UK; ^2^ South West London and St George's Mental Health NHS Trust London UK

**Keywords:** celiac disease, gluten‐free diet management, hypervigilance

## Abstract

**Background:**

Management of celiac disease (CD) requires adherence to a strict gluten‐free diet (GFD). However, little is known about how children with CD manage the GFD. This qualitative study sought to gain a comprehensive understanding of how children with CD experience and navigate the GFD, focusing on their dietary preferences, perceptions, and challenges, as well as the impact of these experiences.

**Methods:**

Fifteen children with CD, aged 8–13 years, who had been following the GFD for a minimum of one year, were interviewed with their parents about their management of the GFD. Reflexive thematic analysis was used to analyze the data.

**Results:**

Children described a range of strategies for managing the GFD. While some adopt problem‐focused strategies such as planning ahead and carrying gluten‐free foods on their person, others exhibit heightened anxiety and persistent doubts, indicating the need for tailored healthcare support. Importantly, the study uncovers socioecological influences, including social roles, communication patterns, and environmental factors, which shape children's beliefs and coping strategies.

**Conclusions:**

The study underscores the importance of monitoring gluten‐related distress, beliefs, and behaviors in children with CD, as well as the broader context of children's lives. To better support children with CD, holistic support may target anxiety to support well‐being alongside GFD management.

**Key message**

This study enhances understanding of how children with celiac disease manage their gluten‐free diet, emphasizing the need for tailored support that addresses both dietary and emotional challenges. The findings provide recommendations for holistic care that are highly relevant to healthcare professionals and researchers in pediatric care.

Celiac disease (CD) is an autoimmune condition that affects approximately 0.9% of children worldwide.^1^, ^2^ Symptoms include diarrhea, constipation, abdominal pain, and weight loss.^3^ In individuals with CD, ingestion of gluten causes a mucosal immune response leading to structural changes in the small intestine, resulting in malabsorption of essential nutrients. The only treatment for CD is lifelong adherence to a gluten‐free diet (GFD). Poor management of a GFD in children with CD increases the long‐term risk of anemia, osteoporosis, infertility, and certain cancers.^3^ GFD adherence demands vigilant oversight during food preparation and scrutiny of food labels.^4^ Adherence poses specific challenges for children, who must navigate the cognitive demands of GFD management,^5^ while transitioning from family management^6^ to increased independence and self‐management.^7^ Only 78% of children achieve optimal adherence, although this depends on the method used to assess adherence, which is problematic as persistently poor adherence negatively impacts health and well‐being.^8^ Compared to children without CD, children with CD have impaired quality of life related to socializing,^9^ with social factors identified as barriers to GFD adherence and contributing to impaired psychological well‐being and quality of life.^10^, ^11^ Some children may intentionally consume gluten to enjoy a desired food, while others may be cautious around their food consumption driven by fears of adverse reactions.^5^ Our current understanding of eating behaviors in CD stems from research with adults, with limited work addressing children's eating behaviors. One exception to this was a mixed methods study, in which 50% of adolescents (13–17 years old) adopted what has been termed “hypervigilant” approaches to GFD management, characterized by preoccupation, rigidity, control, and avoidance.^12^ However, these eating behaviors were assessed using predetermined criteria rather than children's own perspectives. This study aimed to enrich our understanding of how children interact with and experience food, focusing on their management of the GFD.

## 1 METHODS

### 1.1 Study design

This qualitative study was developed in collaboration with families living with CD, which resulted in substantial enhancements to the recruitment materials and interview guide. The interview guide was refined to better reflect the priorities and experiences of children and their families, which included rephrasing questions to make them more comprehensible for children and adjusting the flow of questions to create a more natural and conversational progression. Importantly, this collaboration directed the focus of key questions toward the child to center their voice, ensuring their unique perspectives were prioritized throughout the study.

### 1.2 Participants and recruitment

Inclusion criteria were children (8–16 years) whose parents reported they had a practitioner‐provided diagnosis of CD via blood test or biopsy, who followed the GFD for at least one year prior to study enrollment. Parents were required to take part in the interview alongside their child to provide emotional support, particularly given the sensitive nature of discussing health‐related behaviors. While this involvement offered valuable contextual insights into family dynamics and dietary management practices, deliberate steps were taken to minimize potential parental influence on adolescents' responses, such as directing questions specifically to the adolescent to center their voice during the interview process. Data were collected throughout 2022.

Opportunistic recruitment occurred through advertisements distributed across the online community. Information power was used to guide sampling instead of data saturation, as the latter is more aligned with a realist approach to research.^13^ Information power was repeatedly appraised throughout data collection and deemed sufficiently rich when the researcher developed a thorough understanding of common experiences and nuances.^14^

### 1.3 Procedure and data collection

Participants were provided with detailed information about the study; electronic parental consent and child assent were obtained. No information was provided about the researchers prior to the interview. Semi‐structured interviews were conducted online with HM‐R following a topic guide informed by prior research^15^, ^16^ (Appendix S1), a theoretical model of eating behaviors in adults with CD,^17^ and consultation with families living with CD. Participants were asked about situations or events in which they found managing the GFD challenging, how they managed these situations, and the support they sought. Children attended the interview with a parent, who supported the children in answering interview questions by reminding them of relevant situations. Interviews occurred in a single session and lasted 41.3–92.3 min (mean = 59.2 min). Interviews were video recorded, and field notes were written after interviews. Participants were sent a summary of findings, but feedback was not sought as researcher interpretation plays a central role in reflexive thematic analysis.^18^ Seeking feedback could unintentionally prioritize participants' immediate responses over the depth of interpretation that emerges through the analytical process.

### 1.4 Analysis

Adopting a contextualist epistemological approach, the analysis used reflexive thematic analysis to assess both child and parent experiences. Analysis followed the six phases outlined by Braun and Clarke^18^: familiarizing with the data, coding, generating initial themes, developing and reviewing themes, refining, defining, and naming themes, and writing the report. Data analysis was managed in NVivo; the first three stages were completed by HM‐R, and the latter three by HM‐R, CJ, and RS. Data immersion was achieved through multiple viewings of the interview recordings before analysis. To ensure rigor, the dataset was systematically examined twice during the coding process, and the order of the transcripts was varied to avoid potential bias in the coding. Initially, codes primarily captured semantic meaning, staying true to the participants' language and preserving the nuances in the data (e.g., “live life on guard”). As the analysis progressed, more latent codes were generated to explore underlying themes (e.g., “constant vigilance”). An iterative process of combining and broadening codes was employed to develop potential themes; visual thematic maps and research team discussion were used to refine themes. Pseudonyms were used throughout to ensure anonymity.

### 1.5 Researcher reflexivity

Due to the subjective nature of qualitative methodology, it is important to understand the researcher's positioning. The researchers identify as female and have a doctoral level of psychology training. The interviewer (HM‐R) was a Trainee Clinical Psychologist, which may have facilitated the in‐depth discussions around thoughts and feelings with children. Furthermore, the research team engages in public events focusing on topics such as GFD adherence, well‐being, and food concerns, which may have facilitated discussions surrounding concerns related to food.

### 1.6 Ethics

The study received ethics approval from the Leeds West NHS Research Ethics Committee reference A19/YH/0447.

## 2 RESULTS

### 2.1 Participants

Fifteen children participated in the interviews, alongside their parent (Table 1). Nine children identified as female, six as male. The mean age of the children was 10.5 years, and the mean duration of CD diagnosis was 5.9 years. Three children reported comorbidities alongside their CD diagnosis (lactose intolerance, type 1 diabetes and eczema).

**TABLE 1:** Participant demographic information.

Child pseudonym	Biological sex	Age (years)	Years since CD diagnosis
Alex	Male	13	11
Alfie	Male	11	5
Amelia	Female	8	1
Brogan	Female	9	3
Callum	Male	9	7
Charlotte	Female	8	6.5
Emily	Female	12	8
Grace	Female	12	9
Harriet	Female	11	7
Isaac	Male	8	6
Jodie	Female	13	4
Katie	Female	13	9
Logan	Male	8	2
Martina	Female	10	1
William	Male	12	9

### 2.2 Findings

Two themes were generated: (1) the need for control over others and the environment; and (2) heightened threat and hypervigilance. Despite a broad inclusion criterion, vigilance and hypervigilance around gluten were prominent experiences among participants. Subthemes are in bold within the main text. Table 2 presents a summary of themes with additional illustrative quotes.

**TABLE 2:** Themes and subthemes with illustrative quotes.

Theme	Subtheme	Illustrative Quotes
Theme 1: The need for control over others and the environment	A reliance on others	*“You have no control over what they cook in the kitchen. So like you, you don't know if they could use the wrong spoon or you don't know what they could do with cross contamination”* (Emily) *“Because it's such an, a misunderstood um er disease, a lot of people don't realize how serious it is and how strict it needs to be.”* (Amelia's mother) *“the gluten‐free food was prepared properly and it was put out first. But in that environment, Logan just didn't want, didn't want to risk it”*. (Logan's father)
	Limited food availability and choice	“*I have a very restricted section. Uh, so and sometimes there's basically no food there […] if I didn't like the foods, I wouldn't have any lunch for the day*.” (Alex) *“what if you're a 10‐year‐old girl who has been forced to do this diet and wants something nice and chocolatey for once!”* (Martina) “*They like go out of their way to uh make stuff more accessible for people like vegans, when it's… harder for people who are celiac because uh we if we eat gluten, it affects us*.” (Alex)
	Learning and building trust	“*we don't really have to worry because we've been there lots of times and not once have I been glutened*” (Jodie) *“all it takes is one episode like that, and then your whole trust in in one cook is gone” “not sure we ever really trusted them again, did we?” (Emily's mother)*. “*It's habit that even if you know it's gluten free, it's still kinda that thing of ‘Oh let me just check the ingredients’*” (Jodie).
Theme 2: Heightened threat and hypervigilance	Persistent thoughts about celiac symptoms	“*I think it's worry about potentially what might happen, but he hasn't had any particularly strong reactions so, I think that might have an impact*.” (Callum's mother) *“I get that feeling. I get quite worried and like freak out” (Emily)*
	Imagined series of incidents that could lead to gluten‐contamination	“*Instead of eating, I usually like, I used to think about kind of like all the reas‐ all the reasons I could get ill through cross contamination. It'd just kind of take up my mind*” (Katie)
	Extensive risk‐assessment	“*I can see crumbs falling on the table […] when they pick up like a sandwich or something or a crisp, they might fall on the table like a bit of crumbs […] it's not gonna fall near me without me knowing*” (Amelia) “*I kinda just panic I'm like, OK they're getting closer to me that is getting closer to me and panic goes through my head.”* (Martina) *“it'll be like three days before I decide to kind of like eat one before. Like, every day I check the ingredients. I think it's just that, like, I have to make myself believe that it is gluten free.”* (*Katie*)

### 2.3 Theme 1: The need for control over others and the environment

This theme highlights children's **reliance on others** to manage their GFD, including extended family, friends' parents, and strangers, as well as their need for control over their diet. The balance between these dynamics posed significant challenges, as children expressed apprehensions about entrusting others with the preparation of their gluten‐free foods. These concerns were particularly palpable when food was prepared by unfamiliar individuals or by those who had inadvertently exposed children to gluten previously. A sense of frustration and injustice arose, as children felt others underestimated the importance of the GFD, as William expressed: “*they didn't really care.”* However, children often found solace in relying on trusted others who took steps to minimize the risk of cross‐contact (e.g., through advocating and asking questions around food preparation in restaurants).

Environmental factors, including the proximity of others consuming gluten and the quantity of gluten‐containing foods present, appeared to influence children's perceived control. To cope, children repeatedly checked food labels, consulted with their parents before consuming food provided by others, and advocated for themselves by asking about ingredients and food preparation. In some cases, children even instructed others on appropriate food preparation, as exemplified by Katie when eating at a restaurant: “*we told him* [restaurant staff] *to change their gloves.”* For some, environmental concerns arose due to the developmental context of peers being “*messy” (Harriet)*, grabbing food, and “*throwing food about, and that really got him anxious” (Logan's father)*. In response, children physically distanced themselves from others, asked others to eat more carefully: “*tell her* [sibling] *to use a plate and stop dropping her crumbs” (Grace's mother)*, and sometimes temporarily stopped eating altogether. This behavior was typically observed during specific social events where gluten‐free options were unavailable or where they felt uncertain, rather than a broader, general avoidance of eating. These strategies appeared to enable children to feel more in control of their food, providing a sense of safety. However, they also presented challenges related to social acceptance and straying from peer group norms. For example, Martina describes how advising an adult may be perceived as disrespectful: *“you can't do that as a child cos sometimes you just get [told] you're being cheeky and rude” (Martina)*.

**Limited food availability and choice** of gluten‐free food exacerbated children's sense of control. While gluten‐free snacks were more readily available, securing proper meals was challenging. The “*repetitive*” *(Emily)* and “*boring” (Katie)* gluten‐free selections left many feeling dissatisfied, while some became “*protective” (Callum's mother)* of their food to regain a sense of control. Children questioned why they had to contend with such limited choices, explaining this was “*unfair” (Emily)* and questioning “*why me?” (Grace)*. Many highlighted the disparity between support offered to lifestyle diets and the insufficient attention given to the seriousness of CD. To cope with these obstacles, children often took their own food to school and social events, and planning and preparation (e.g., checking restaurant reviews for gluten‐free options) became essential to ensure a safe and enjoyable dining experience, often managed by parents.

Participants' accounts also revealed a process of **learning and building trust**, to better manage the GFD. When gluten cross‐contact occurred, children tried to make sense of this, struggling when symptoms were experienced with no known cause: “*that unknown, is very hard psychologically to get your head round” (Emily)*. Learning from past experiences of cross‐contact, children adapted their behaviors to reduce future risks, such as asking questions about food. Building trust in others and food products was vital to feel safe when dining out, however a single incident of cross‐contact could fracture this trust: *“all it takes is one episode like that, and then your whole trust in one cook is gone*” *(Emily's mother)*. For some younger children, parents took responsibility for managing the GFD, resulting in fewer concerns about cross‐contact from the children: “*I basically just do what you say I can eat” (William)*. Parents and children recognized that their experience of managing the GFD might change with age and independence, with some anticipating increased difficulties, while others expected improvements. There appeared to be a process of adjustment to the GFD, and most children stated they *“got used to it” (Jodie)* over time. Learning from experiences helped children in developing a problem‐focused approach to managing the GFD that became habitual: “*Now it's habit that even if you know it's gluten free, it's still kinda that thing of ‘Oh let me just check the ingredients’” (Jodie)*, reflecting a pragmatic approach to dietary management.

### 2.4 Theme 2: Heightened threat and hypervigilance

Throughout children's narratives was a sense of fear around gluten cross‐contact, with children referring to gluten as “*dirty” (Brogan)* or “*bad” (Isaac)*. Children expressed **persistent thoughts about celiac symptoms**: *“I used to think about all the reasons I could get ill through cross contamination. It'd just kind of take up my mind” (Katie)*. The type of symptoms children experience seemed to influence the intensity of their concerns, with those experiencing gastrointestinal symptoms expressing more concerns: “*I'm scared of the throwing up (Brogan)*.” In contrast, Callum's mother referred to Callum's lack of “*strong reactions*” in explaining his lack of worries. Persistent thoughts around gastrointestinal symptoms appeared to be present when eating, although parents noted that their children may experience difficulty distinguishing between symptoms caused by gluten cross‐contact and those induced by anxiety itself, as reported by Grace's mother: “*At some point you can't quite tell which one's gluten.”*

To prevent exposure to gluten, many children **imagined scenarios that could lead to gluten contamination**, particularly in settings outside of their control. As Martina describes:

“There's obviously a big chain like where you touch one [gluten‐containing item] and then you could go eat and you're still touching that thing, and then you touch your food, and then that food goes into your mouth, and then you get glutened. So just a big long food chain of stuff that sometimes doesn't even make sense.”

This analytical process appears to be driven by a heightened sense of threat around being exposed to gluten. The language used by some children further evidenced this, appearing to reflect a process in which they thought in extremes, such as: *“literally everything was contaminated”* and “*all the utensils will have had some form of contamination on*” *(Jodie)*.

To prevent gluten cross‐contact, children engaged in **extensive risk assessments**, including constantly evaluating their surroundings, assessing crockery cleanliness, and closely observing food preparation. A particular source of concern was eating near peers and being contaminated by their food, resulting in some children actively watching others eat and distancing themselves to avoid exposure. Some adopted strategies of physically protecting their food by positioning their lunchbox away from peers or placing their arms around their food, as described by Amelia: “*I have, like, tin foil or something, I push up the edge of it so nothing will go like past it, yeah. So then I make a little like wall around it*.” Some children had particular difficulty trusting new foods or eating in new settings, resulting in them checking for gluten content multiple times despite reassurances. For many, this fear led to avoidance of certain foods, restaurants, or social events, and for some, led to restricting eating: *“Usually on airplanes I choose to starve myself” (Brogan)*. However, some children appeared flexible and pragmatic in their approach, despite feeling cautious. These children adopted self‐management strategies to manage potential risks: “*I'll make him a packed lunch to take” (Logan's father)*, “*I don't really mind touching* [gluten‐containing foods] *because I know if I fully wash my hands then I'll be alright” (Charlotte)*.

## 3 DISCUSSION

While research suggests variation in the approaches adults take to manage the GFD can impact quality of life and well‐being,^14^, ^15^, ^19^ little is known about children's approaches to managing the GFD. This study used robust qualitative methods to explore how children with CD experience and navigate the GFD.

Children described a range of approaches to manage the GFD. Pragmatic approaches were characterized by children's acceptance of the dietary restrictions with an awareness of cross‐contact and potential consequences. Despite reported concern around cross‐contact, these children took practical steps (e.g., planning ahead, taking gluten‐free snacks, and asking questions around food preparation) to manage the potential health threats, which they applied flexibly, enabling them to engage in social activities. These strategies appear to reflect a problem‐focused coping approach, an effective approach for supporting well‐being and dietary management in children with food allergies.^20^ This approach appears similar to the eating behaviors described by adolescents and adults with CD, which are associated with improvements in quality of life.^12^ Conversely, hypervigilant approaches were characterized by heightened perception of threat, persistent thoughts about CD symptoms, and preoccupation about potential gluten cross‐contact. Consistent with Cadenhead et al.,^12^ these children coped with concerns about cross‐contact by engaging in extensive risk‐assessments, restricting eating, and avoidance of social activities involving food. Crocco et al.^21^ demonstrated no change in the quality of life for children with CD over 10 years, yet they recommend ongoing monitoring of patients' quality of life and the development of diverse assessment tools to capture its nuances. These findings suggest that assessing hypervigilance may be an important component of quality of life that should be incorporated into evaluation measures for children with CD.

The dietary approaches described here appeared to have a psychological impact on children, consistent with previous research.^10^ This analysis provides new insights into the complexities faced by children regarding potential threats from gluten exposure. Despite asking a broad range of questions around dietary management, children in this study maintained a focus on the threat of gluten exposure. Hypervigilance and selective attention to threats are known to trigger and perpetuate anxiety,^22^ and in some children, this selective attention to gluten was apparent (e.g., mentalizing steps to gluten cross‐contact, protecting foods with physical barriers). Anxiety, which is more prevalent in adults with mucosal healing, is believed to be mitigated by a more vigilant approach to GFD adherence.^23^ For adults with CD, it has been proposed that an overestimation of the likelihood of gluten cross‐contact and a perceived lack of control contribute to fear and anxiety.^17^ Similarly, children here described persistent thoughts about symptoms, a preoccupation with potential incidents leading to cross‐contact, and a perceived lack of control, which appeared to exacerbate anxiety. In attempting to manage the health threat, children employed safety behaviors such as a hypervigilant focus on food preparation and extensive risk assessments.

The experiences of children underscore the profound impact of sociocultural factors on GFD management, extending beyond what is observed in adults.^24^ Contextual influences shape beliefs regarding gluten cross‐contact risk, as well as the subsequent appraisal and anxiety levels among children. Sociocultural factors, including social roles and communication patterns, may play a pivotal role in these perceptions, especially considering children's sensitivity to social hierarchy cues.^25^ Children often view adults as authoritative figures in a position of power to advise children, leading to discomfort when children are required to behave contrary to their social role by advising adults on food preparation. Societal expectations around authority and respect can shape children's willingness to advocate for their dietary needs. This builds on existing literature that negative power dynamics with medical professionals limited disclosure of psychological difficulties in adolescents with CD,^26^ underscoring the influence of power on communication challenges in children and adolescents with CD. Similarly, Skjerning and colleagues^5^ highlight the interplay between context and coping, illustrating the decision‐making process for children in social situations involving food. This highlights the necessity of adapting current understandings of GFD management, particularly hypervigilant approaches, to incorporate the overarching influence of context. This entails considering developmental factors, such as supporting the transition to independent GFD management, as well as social influences, environmental conditions, structural aspects such as food availability, and biological responses to symptoms. Recognizing and addressing these sociocultural aspects is crucial for developing comprehensive support systems tailored to the diverse needs of children managing CD and their families.

### 3.1 Strengths and limitations

A notable strength of this study lies in its representation of age and biological sex across children; however, this study was not designed to compare perceptions by biological sex or age. We acknowledge that these factors could influence experiences and may be an avenue for further work. Furthermore, the absence of information about participants' ethnicity hinders the analysis of cultural influences on children's GFD management. This study obtained children‐informed perspectives about GFD management, a topic previously informed by predominantly adult theory. However, children were interviewed alongside their parent, which might have limited their disclosure of instances whereby they inadvertently or intentionally consumed gluten. Consequently, they might have felt more at ease discussing strict adherence to the GFD. Previous research indicates that younger children, such as those in this study, tend to adhere more strictly to a GFD than adolescents and that their relationship with the GFD may evolve over time.^27^

It is also important to note that the diagnosis of CD was parent‐reported. While we required confirmation through blood tests and biopsy, there remains a possibility that some children may not have CD despite parental report. Additionally, this may include those who are not receiving adequate healthcare support around the GFD, which could contribute to increased anxiety. However, since most children with CD are managed within the community, we believe this sample provides a good representation of the experiences of children living with this condition.

### 3.2 Clinical and research implications

Improved support for children with CD is essential due to its significant impact on well‐being and quality of life. Both pediatric and specialist services should focus on developing problem‐focused coping strategies, such as planning and managing situations with potential gluten cross‐contact. Training in communication skills can empower children to assertively express their dietary needs, which may help them navigate challenging social dynamics.

There is a notable research gap in evidence‐based interventions for children experiencing distress related to CD. Both dietetic and psychological support show promise in addressing these concerns, including Cognitive Behavioral Therapy approaches incorporating techniques such as cognitive restructuring, worry management, and mindfulness.^15^ Many children had a heightened perception of risk in certain situations, such as dining near others who consume gluten; this hypervigilance may stem from inadequate education provided to patients and families during diagnosis and follow‐up. Evidence indicates that improved educational efforts can enhance coping mechanisms and alleviate unnecessary anxiety surrounding food interactions.^28^, ^29^ However, current clinical practices in Europe may not fully address these needs.^30^

Many children in the study were diagnosed at a young age, raising concerns about their understanding of the GFD. If they primarily rely on parental guidance, their approach to the GFD could be significantly shaped by their parents' attitudes, including levels of hypervigilance. This prompts the question of whether such hypervigilance may be “transferred” from parent to child, particularly among younger participants. Future research should investigate how gluten‐related beliefs evolve over time, especially during the transition to self‐management in adolescence, and whether levels of gluten preoccupation change with prolonged adherence to the GFD. A deeper understanding of these dynamics could enhance the management and support provided to children with CD.

## 4 CONCLUSIONS

The study highlights a continuum of GFD management approaches among children with CD, ranging from pragmatism to hypervigilance. Practical strategies adopted and applied flexibly by children reflect problem‐focused coping, which is crucial for their well‐being and dietary management. On the other hand, hypervigilant approaches were accompanied by heightened anxiety, persistent doubt regarding gluten exposure, and avoidance, likely impairing quality of life.

### Significance of this Work

While research has primarily focused on adults' strategies for managing the gluten‐free diet (GFD) for celiac disease (CD), there is limited understanding of how children manage the GFD from their own perspective. This study fills that gap by exploring children's approaches, revealing a continuum of management strategies from pragmatic, problem‐focused coping to hypervigilant approaches often linked to heightened anxiety. Findings emphasize the importance of tailored healthcare support to enhance quality of life and well‐being in children with CD.

## FUNDING INFORMATION

This research received no specific grant from any funding agency, commercial, or not‐for‐profit sector.

## CONFLICT OF INTEREST STATEMENT

The authors declare no conflicts of interest related to this study.

## Supporting information


**Data S1:** Interview guide.
