# Supplementary material for: Gluten‐free diet management and well‐being in children with celiac disease: A qualitative study
Source: Pediatr Allergy Immunol. 2025 Mar 18;36(3):e70061. doi: 10.1111/pai.70061 (PMC11916637; doi:10.1111/pai.70061)
Supplement: Supplementary file 1 — Data S1: Interview guide. [file PAI-36-e70061-s001.docx]

Appendix 1: Interview Guide

Introductory question:  Let’s start the discussion by talking about your coeliac disease diagnosis. What happened and what was that like for you both?

Key question #1: Think about your daily life with coeliac and adhering to a gluten-free diet, what challenging situations do you encounter, particularly around managing cross contamination? *Inside/outside home; peers; family*

Key question #2: Over the past few months, what situation was most challenging for you to manage cross contamination, and how did you deal with it? *Inside/outside home; conflict; peers; daily annoyances and challenges?*

Key question #3: Let’s choose a particular situation, where you felt worried about cross-contamination. How did you feel and what did you do to manage these concerns? *Specific symptoms, meanings and appraisals as well as emotional and behavioural responses.*

Key question #4: Think about experiences in or out of the home environment, what do you find helpful or supportive/ restrictive? *Parental support; independence; labelling; asking staff; your own credibility in disease management*

Key question #5: Now let’s think about the support you receive in clinic to try and address these types of concerns around cross-contamination. What type of help and support would be useful as part of your treatment plan?

Summary question: How well did this discussion capture what it is like living with celiac disease and managing cross-contamination?

Final question: Is there anything that we should have talked about but didn't?
